# Left Atrial Primary Leiomyosarcoma Presenting With Complete Right Pulmonary Vein Thrombosis in a Young Male Patient: A Case Report and Literature Review

**DOI:** 10.7759/cureus.92919

**Published:** 2025-09-22

**Authors:** Dumitru Cravcenco, Marinela Secrieru, Maria Cobzac, Feodor Ostopovici

**Affiliations:** 1 Radiology, Scanexpert Medical Diagnostic Center, Chisinau, MDA; 2 Radiology, Nicolae Testemițanu State University of Medicine and Pharmacy, Chisinau, MDA

**Keywords:** cardiac sarcoma, case report, left atrium, leiomyosarcoma, pulmonary vein thrombosis, young male

## Abstract

Primary cardiac leiomyosarcomas are extremely uncommon, constituting only a very small fraction of all primary cardiac tumors and a minor subset of cardiac sarcomas. Nearly half occur in the left atrium due to smooth muscle elements in the pulmonary venous junction. Their nonspecific presentation, often mimicking atrial myxoma or thrombus, delays diagnosis and treatment. A 19-year-old male patient presented with recurrent hemoptysis and exertional dyspnea. Initial workup suggested pneumonia; however, contrast-enhanced computed tomography (CT) and echocardiography revealed a large, heterogeneous mass (7.0 × 4.5 × 6.0 cm) filling the left atrium with complete right pulmonary vein thrombosis. Urgent open-heart surgery achieved en bloc tumor excision, posterior left atrial wall reconstruction, right pulmonary vein reimplantation, and valve repairs. Histopathology confirmed an intermediate-grade leiomyosarcoma (French Federation of Cancer Centres grade 2) with spindle cells, necrosis, mild atypia, five mitoses per 10 high-power field, and smooth muscle actin/vimentin positivity. Postoperative CT demonstrated a tumor-free reconstructed atrium and patent pulmonary vein. Complete pulmonary vein thrombosis is a rare but critical indicator of malignant cardiac tumors. While multimodal imaging (echocardiogram, cardiac magnetic resonance imaging, CT, and positron-emission tomography) assesses tumor extent, histology and immunohistochemistry remain diagnostic standards. According to the literature, the prognosis remains poor, and survival rates are low even when surgery is combined with adjuvant chemotherapy. Rare long-term survivors are reported after multimodal treatment. An atrial mass with complete pulmonary vein thrombosis strongly suggests malignancy. Early surgery, histological confirmation, and multidisciplinary care are essential, although prognosis remains poor.

## Introduction

Primary cardiac tumors are exceptionally uncommon, with an incidence estimated between 0.0017% and 0.03% in autopsy series [1]. Approximately three-quarters of these lesions are benign, most commonly myxomas, while 25% are malignant, with sarcomas accounting for the majority [1,2]. Primary cardiac leiomyosarcomas (PCLMS) represent an exceedingly rare subset, comprising less than 0.25% of all primary cardiac tumors and approximately 8%-9% of cardiac sarcomas [3]. Nearly half of PCLMS originate in the left atrium, often associated with the smooth muscle-rich junction of the pulmonary veins [1,3]. These tumors predominantly affect middle-aged adults (mean age 45-50 years) and exhibit a slight female predominance; these tumors are unusual in a 19-year-old male patient, making this case particularly uncommon [1,4].

Clinically, PCLMS often present insidiously, with nonspecific symptoms such as exertional dyspnea, orthopnea, hemoptysis, chest discomfort, syncope, or signs of congestive heart failure [5-7]. These manifestations frequently mimic benign cardiac masses such as atrial myxomas or organized thrombi, resulting in diagnostic delays and initial mismanagement. Advanced disease is frequently diagnosed only when complications occur, including pulmonary hypertension, severe obstruction of the mitral valve or pulmonary veins, or systemic embolization [3,8]. Imaging modalities such as transthoracic and transesophageal echocardiography, cardiac magnetic resonance imaging, computed tomography (CT), and positron-emission tomography (PET) provide critical anatomic and functional details, yet definitive diagnosis relies on histopathological confirmation [1,9,10]. Leiomyosarcomas typically exhibit spindle-shaped cells with marked pleomorphism, necrosis, elevated mitotic activity, and immunopositivity for smooth muscle markers such as smooth muscle actin (SMA), caldesmon, and vimentin; the Ki-67 proliferation index frequently exceeds 50% in high-grade tumors [7,11].

This case is distinctive because it documents a primary left atrial leiomyosarcoma presenting with complete thrombosis of the right pulmonary veins, an exceptionally rare manifestation that strongly indicates aggressive malignant behavior [3,4]. Such vascular involvement is seldom seen in benign cardiac lesions and serves as a radiological hallmark of malignancy.

The objective of this report is to present a rare clinical case, underscore the diagnostic relevance of pulmonary venous obstruction as an indicator of malignancy, and review the current literature regarding the clinical features, diagnostic approach, and management strategies for PCLMS. This case highlights the necessity of early recognition, prompt surgical intervention, and multidisciplinary management to improve outcomes in this aggressive disease.

## Case presentation

A 19-year-old male patient, with no significant past medical history, presented in September 2024 with acute hemoptysis and progressive exertional dyspnea. He denied prior cardiovascular disease, trauma, or systemic symptoms such as weight loss or fever. On initial evaluation, he was afebrile and hemodynamically stable, but exhibited mild hypoxemia and decreased breath sounds at the right lung base. Laboratory studies revealed mild anemia (hemoglobin 11.4 g/dL), normal inflammatory markers, and normal renal and hepatic function.

The patient was admitted with a presumed diagnosis of right lower lobe pneumonia and initiated on intravenous antibiotics. Despite therapy, hemoptysis persisted, accompanied by pleuritic chest pain and worsening dyspnea, prompting transfer in January 2025 to a tertiary care center for further evaluation.

**Figure 1:**
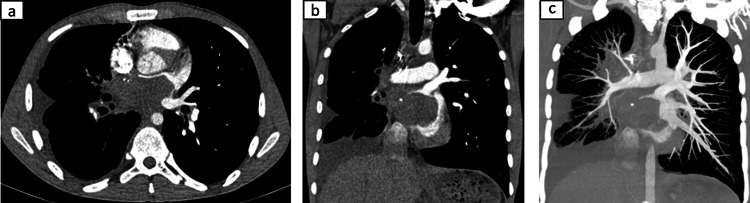
(a) Axial CT. (b) Coronal CT. (c) Coronal CT, MIP (a,b) Contrast-enhanced CT of the chest revealed a large, lobulated, nonenhancing heterogeneous mass occupying nearly the entire left atrial cavity. (c) Complete thrombosis of the right superior and inferior pulmonary veins CT: computed tomography; MIP: maximum intensity projection

Contrast-enhanced CT of the chest revealed a large, lobulated, nonenhancing heterogeneous mass measuring 7.0 × 4.5 × 6.0 cm occupying nearly the entire left atrial cavity (Figures 1a, 1b). The lesion infiltrated the posterior atrial wall and was associated with complete thrombosis of the right superior and inferior pulmonary veins, extending to their junction with the left atrium (Figure 1c). There was no evidence of distant metastases. Transthoracic and transesophageal echocardiography demonstrated a hyperechoic, immobile mass (35 × 53 mm) within the left atrium, with grade II mitral and tricuspid regurgitation and severe pulmonary hypertension (estimated pulmonary artery systolic pressure ~65 mmHg). No blood flow was detected within the mass on Doppler interrogation.

**Figure 2:**
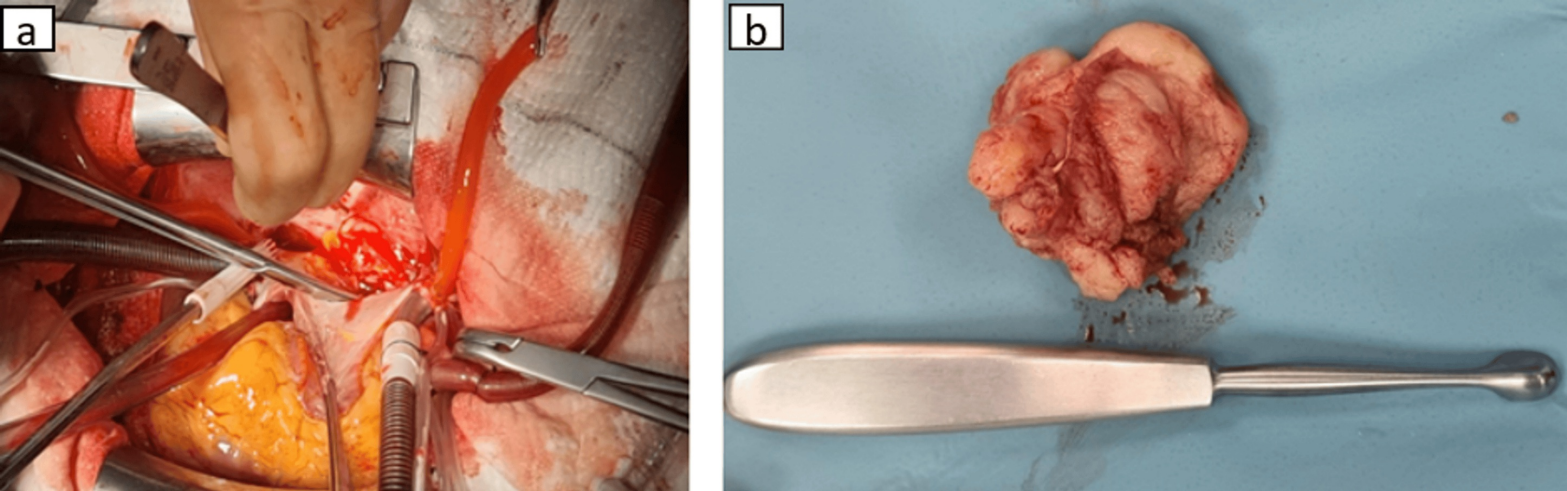
Operative findings revealing (a) a solid tumor arising from the posterior wall of the left atrium. (b) The mass was excised en bloc

Given the high suspicion for malignancy due to the extensive venous involvement, the patient underwent urgent open-heart surgery in February 2025. Intraoperative findings revealed a solid tumor arising from the posterior wall of the left atrium, extending to the confluence of the right pulmonary veins. The mass was excised en bloc, followed by reconstruction of the posterior left atrial wall and reimplantation of the right pulmonary veins into the neoatrium, interatrial septum plasty, right atrial plasty using a bovine pericardial patch, mitral valve repair, and De Vega-Cabrol tricuspid annuloplasty (Figures 2a, 2b).

Histopathologic examination showed intersecting fascicles of spindle cells with mild nuclear atypia, areas of necrosis, and five mitoses per 10 high-power fields. Immunohistochemical staining was positive for SMA and vimentin, while negative for myogenin and desmin. The findings supported the diagnosis of intermediate-grade primary cardiac leiomyosarcoma (French Federation of Cancer Centres grade 2).

**Figure 3:**
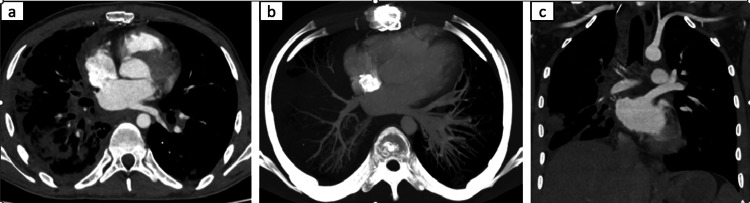
(a) Axial CT. (b) Axial CT, MIP. (c) Coronal CT On follow-up contrast-enhanced CT postoperatively, (a,c) the reconstructed left atrium appeared normal, with no evidence of residual tumor, and (b) the reimplanted right pulmonary vein CT: computed tomography; MIP: maximum intensity projection

On follow-up contrast-enhanced CT postoperatively, the reconstructed left atrium appeared normal, with no evidence of residual or recurrent tumor (Figures 3a, 3c). The reimplanted right pulmonary vein was patent, with normal opacification and no filling defects (Figure 3b). The patient remained clinically stable, with resolution of hemoptysis and improvement in exercise tolerance. Adjuvant chemotherapy was planned with a doxorubicin-ifosfamide regimen, and the patient was scheduled for serial clinical and imaging follow-up.

## Discussion

PCLMS represent a particularly rare subset of primary cardiac malignancies, accounting for less than 0.25% of all primary cardiac tumors and only 8%-9% of cardiac sarcomas [1,3,4]. Epidemiological data from the French Sarcoma Group, the largest multi-institutional cohort to date, encompassing 124 patients with primary cardiac sarcomas, demonstrate a median age of 48.8 years, a slight female predominance (56.5%), and a median overall survival of 17.2 months across all subtypes [1]. Leiomyosarcomas were predominantly left-sided (75%) and located in the atria [1]. Our case is unusual not only because it involves a teenage boy, far younger than the typical demographic, but also due to the extensive venous involvement, with complete thrombosis of the right pulmonary veins, an exceptionally rare presentation rarely documented in benign neoplasms [3,4].

Diagnostic challenges and differential considerations

The clinical presentation of PCLMS is typically nonspecific and overlaps significantly with benign cardiac conditions. Common symptoms include exertional dyspnea, orthopnea, chest discomfort, palpitations, hemoptysis, and features of heart failure [5-7]. These nonspecific manifestations often lead to initial misdiagnosis, frequently as pneumonia, pulmonary embolism, or atrial myxoma. The latter is the most frequent benign atrial tumor, and its echocardiographic appearance, mobile, pedunculated, and sometimes myxoid, can closely mimic sarcoma [6]. However, unlike myxomas, leiomyosarcomas typically present as sessile or infiltrative masses with irregular contours, heterogeneous composition, and, in aggressive cases, invasion of adjacent structures, including pulmonary veins [3,4].

Venous obstruction, particularly complete thrombosis of the pulmonary veins, is an uncommon feature in benign tumors and strongly suggests malignancy [3,4]. Both Hrdinova et al. and Malyshev et al. highlighted that involvement of the pulmonary venous confluence or extension into the pulmonary vasculature is a radiological hallmark of sarcoma rather than myxoma [2,3]. In our patient, this finding, combined with the immobility and heterogeneous structure of the mass on echocardiography, prompted expedited surgical intervention despite the absence of prior cardiovascular pathology.

Role of imaging and histopathology

Multimodal imaging plays a critical role in evaluating tumor characteristics, defining the extent of invasion, and planning resection. Transthoracic and transesophageal echocardiography remain first-line tools for identifying intracardiac masses, while cardiac MRI and CT provide complementary information on tissue characterization and extracardiac spread [1,9]. PET can assist in detecting metabolic activity and staging by excluding systemic metastases. However, as emphasized by multiple case series and reviews [1,3,4,7], imaging alone cannot reliably differentiate between benign and malignant atrial tumors. Histological and immunohistochemical evaluation remains the diagnostic gold standard. Leiomyosarcomas typically exhibit intersecting fascicles of spindle-shaped cells with nuclear pleomorphism, necrosis, a variable mitotic index, and immunopositivity for SMA, caldesmon, and vimentin [7,11]. The Ki-67 index, frequently >50% in high-grade cases, reflects the aggressive proliferative nature of these tumors [7].

Therapeutic strategies and outcomes

Complete surgical resection remains the cornerstone of therapy for PCLMS [1,8]. In the French Sarcoma Group cohort, complete (R0) resection was associated with a median survival of 38.8 months, compared to 18.2 months for incomplete resection and only 11.2 months in nonresected patients [1]. However, complete resection is often technically challenging due to the tumor's infiltrative growth and involvement of critical structures such as the pulmonary veins, atrial walls, or mitral annulus. In our patient, en bloc excision was feasible as the mitral valve was uninvolved, allowing for reconstruction of the left atrial wall and reimplantation of the right pulmonary veins.

Adjuvant chemotherapy, typically doxorubicin- and ifosfamide-based, is commonly reported in clinical series, though its impact on survival remains uncertain. Some reports suggest modest benefits in nonresected patients or those with incomplete resections [1,12], while others highlight anecdotal cases of prolonged survival when combined with radiotherapy [12-15]. Heart transplantation has been proposed for select patients with localized, unresectable disease, but remains controversial given the risks of recurrence under immunosuppression and limited donor availability [1].

Despite aggressive management, the prognosis of PCLMS remains poor. Median survival ranges from 12 to 24 months in most case series, even with R0 resection and adjuvant therapy [1,8]. Only a few long-term survivors have been reported, with Nakashima et al. [13] describing a case achieving 27 months of survival following surgery and chemotherapy. Our patient, given his young age, absence of metastatic disease, and successful complete resection, may fall into a subset with a comparatively better prognosis, but long-term outcomes remain guarded.

Clinical implications and limitations

This case underscores several key considerations for clinical practice. First, in any young patient presenting with unexplained hemoptysis, persistent dyspnea, and imaging evidence of a left atrial mass, malignant cardiac neoplasms, though rare, should be considered. Second, complete pulmonary vein thrombosis is an important imaging clue that should heighten suspicion for sarcoma and expedite surgical consultation. Third, successful management relies on a multidisciplinary approach involving cardiology, cardiothoracic surgery, pathology, and oncology to optimize outcomes.

The limitations of this case include the absence of long-term follow-up data and the inability to assess the impact of adjuvant chemotherapy on overall survival at this stage. Nonetheless, its rarity, the patient's unusually young age, and the exceptional finding of complete pulmonary venous thrombosis highlight the importance of reporting such presentations. These observations expand the current understanding of PCLMS and may help clinicians recognize malignant features early, prompting timely and life-saving interventions. To exclude tumor recurrence, dynamic evaluation by contrast-enhanced CT is recommended after completion of the combined treatment.

## Conclusions

Primary cardiac leiomyosarcoma is an exceptionally rare and aggressive malignancy, most frequently arising in the left atrium and often presenting with nonspecific symptoms that mimic benign cardiac masses. Complete thrombosis of the pulmonary veins, as seen in this case, is an extraordinary finding and a critical imaging hallmark that strongly suggests malignant behavior. Early recognition of such atypical features is vital to expedite diagnosis and surgical intervention. Despite complete resection and planned adjuvant therapy, the prognosis remains guarded, with median survival typically ranging from 12 to 24 months. This case highlights the importance of multimodal imaging, histopathologic confirmation, and a multidisciplinary treatment strategy to optimize patient outcomes and underscores the need for further research to refine diagnostic and therapeutic approaches for this rare cardiac sarcoma.
